# Public health for the people: participatory infectious disease surveillance in the digital age

**DOI:** 10.1186/1742-7622-11-7

**Published:** 2014-06-20

**Authors:** Oktawia P Wójcik, John S Brownstein, Rumi Chunara, Michael A Johansson

**Affiliations:** 1 Harvard Medical School and Boston Children's Hospital, 1 Autumn St., Boston, MA 02215, USA; 2 Centers for Disease Control and Prevention, San Juan, Puerto Rico, USA

**Keywords:** Dengue, Influenza-like illness, Participatory surveillance, Participatory surveillance system, Disease surveillance, Public health

## Abstract

The 21^st^ century has seen the rise of Internet-based participatory surveillance systems for infectious diseases. These systems capture voluntarily submitted symptom data from the general public and can aggregate and communicate that data in near real-time. We reviewed participatory surveillance systems currently running in 13 different countries. These systems have a growing evidence base showing a high degree of accuracy and increased sensitivity and timeliness relative to traditional healthcare-based systems. They have also proven useful for assessing risk factors, vaccine effectiveness, and patterns of healthcare utilization while being less expensive, more flexible, and more scalable than traditional systems. Nonetheless, they present important challenges including biases associated with the population that chooses to participate, difficulty in adjusting for confounders, and limited specificity because of reliance only on syndromic definitions of disease limits. Overall, participatory disease surveillance data provides unique disease information that is not available through traditional surveillance sources.

## Background

Community engagement has long been an important part of public health. In the 1850s, John Snow identified the role of water in cholera transmission using data that he acquired by talking with people living in a cholera epidemic [1]. When large smallpox outbreaks diminished as part of the smallpox eradication campaign, the World Health Organization turned to field workers armed with pictures of smallpox victims to survey villagers and find cases of the disease [2]. This approach was critical for identifying the last bastions of disease leading to smallpox eradication [2]. In the veterinary field, a similar effort was carried out in the last stages of the Rinderpest eradication campaign, with farmers identifying cases in their own cattle [3]. This approach was coined “participatory surveillance”, referring to the participation of the community in disease surveillance.

In the 21^st^ century, public engagement is being transformed through participatory surveillance systems that enable the public to directly report on diseases via the Internet. These systems encourage the regular, voluntary submission of syndromic, health-related information by the general public using computers or smartphones. Reported data are aggregated and visualized in near real-time allowing immediate feedback to users and public health agencies. This offers the opportunities to improve disease surveillance by providing data faster and to engage the public by communicating findings directly via the Internet.

In this review, we describe: 1) a selection of active participatory surveillance systems for infectious diseases (Influenzanet, FluTracking, Reporta, Flu Near You, Dengue na Web, and SaludBoricua), 2) their unique contributions to public health, 3) the strengths and weaknesses of participatory surveillance systems for infectious diseases, and 4) the future of participatory surveillance.

### Active participatory surveillance systems for infectious diseases

Internet-based participatory surveillance has only emerged in the last decade. While there are not many systems currently in place, there are new implementations almost every year. The first system, de Grote Griepmeting, or the Great Influenza Survey, was started by a group of individuals from various institutes during the 2003/2004 influenza season in the Netherlands and in Dutch speaking Flanders in Belgium [4]. De Grote Griepmeting was created to monitor the activity of influenza-like illness (ILI) by collecting symptom data from voluntary participants. Participants register, complete an intake questionnaire containing various medical, geographic and behavioral questions, and receive weekly emails for reporting symptoms (or a lack thereof) since their last visit to the website [5] (Table 1). The incidence of ILI among participants is determined in real time using a syndromic case definition [5] (Table 2) and graphic representation of the results is dynamically updated on the system’s web site.

**Table 1:** List of symptoms collected by Influenza-like Illness (ILI) tracking systems: Influenzanet, FluTracking, Reporta and Flu Near You

**Symptom**	**Influenzanet**^**a**^	**FluTracking**	**Reporta**	**Flu Near You**
Fever	✓	✓	✓	✓
Cough	✓	✓	✓	✓
Sore throat	✓		✓	✓
Shortness of breath	✓		✓	✓
Chills/night sweats	✓			✓
Fatigue	✓			✓
Nausea/vomiting	✓		✓	✓
Diarrhea	✓		✓	✓
Body aches/Muscle pain	✓		✓	✓
Headache	✓		✓	✓
Runny or blocked nose	✓		✓	
Sneezing	✓			
Chest pain	✓			
Loss of appetite	✓			
Colored sputum/phlegm	✓			
Watery/bloodshot eyes	✓			
Stomach ache	✓			
Irritation			✓	
Joint pain			✓	
Weakness			✓	

^a^Influenzanet has an extensive symptom questionnaire and not all of the information collected by this questionnaire is listed in Table 1. A complete list of questions can be obtained from the “Methods” section of influenzanet.eu.

**Table 2:** Influenza-like Illness (ILI) definitions for Influenzanet, FluTracking, Reporta and Flu Near You/SaludBoricua

**Participatory surveillance system**	**Influenza-like illness definition**
Influenzanet	Sudden onset of symptoms AND at least one of the following: fever and/or chills, feeling tired or exhausted, headache, muscle pain; AND at least one of the following: cough, sore throat, shortness of breath
FluTracking	Fever AND cough
Reporta	Fever AND at least one of the following: cough or sore throat
Flu Near You/SaludBoricua	Fever AND at least one of the following: cough or sore throat

The idea of Internet-based participatory ILI surveillance spread quickly and the model was adopted by other European countries [6]: Portugal (Gripenet started in 2005 [7]), Italy (Influweb started in 2007 [8]), United Kingdom (Flusurvey started in 2009 [9]), Sweden (Influensakoll started in 2011), France (Grippenet started in 2012), and Spain (Gripenet started in 2012 [5]). These systems have been supported by private individuals, numerous national agencies and foundations, as well as the European Commission and are now collectively called Influenzanet. Collaboration between these systems has led to standardization of collection and analytical methods which allows comparability between countries.

A similar participatory ILI surveillance system, FluTracking, was launched in Australia in 2006 as a joint project between the University of Newcastle and Hunter New England Health [10]. FluTracking started with a focus on south-eastern Australia, but expanded nationally in 2007 [11]. Like Influenzanet, participants are asked to report weekly and data is published online as a map and a newsletter at the end of each week. FluTracking also focuses on examining the yearly effectiveness of the influenza vaccine using its data [12].

Based on Portugal’s Gripenet, Reporta was launched in 2009 in Mexico to track respiratory disease, including ILI. The system was initially created in the Mathematical Visualization Laboratory with funding from the Institute of Science and Technology of Mexico and the Center for Complexity of Science at the National Autonomous University of Mexico [13]. Like the other systems, Reporta collects symptom data from residents on a weekly basis and the website displays data in real-time together with other important news and information about influenza and public health in Mexico.

Flu Near You is a U.S. based system developed by HealthMap at Boston Children’s Hospital, the Skoll Global Threats Fund, and the American Public Health Association (APHA) and launched in 2011 [14]. Flu Near You allows individuals to register using its website, mobile application or Facebook. Like the other systems, Flu Near You targets ILI and collects data on a weekly basis, which it publishes in real time, while offering a user interface to compare its data with data from Centers for Disease Control and Prevention (CDC) sentinel influenza network and Google Flu Trends. Additionally, Flu Near You also hosts a “Vaccine Finder” which allows individuals to identify local sources for influenza vaccination [14].

Dengue na Web was the first system to target non-respiratory diseases. It was launched in 2011 to monitor dengue activity in the city of Salvador de Bahia, Brazil. Inspired by the Gripenet project, Dengue Na Web was developed by the Federal University of Bahia with support from various state and federal entities [15]. Dengue na Web offers real-time maps of participant reports along with educational materials and international news about dengue research.

In November 2012, SaludBoricua, an expanded version of the Flu Near You system, was launched in Puerto Rico. While the reporting mechanisms and data display are virtually identical to Flu Near You, SaludBoricua is unique among the other systems because instead of targeting a single disease, it targets three different acute febrile illnesses: influenza, dengue and leptospirosis [16].

While system design and objectives vary across these systems, they all include a registration process which collects varying amounts of background data and weekly email prompts to encourage reporting of symptoms experienced that week (Table 1). Symptom data is processed in real-time using syndromic definitions (Table 2) and is displayed, generally in the form of maps or timelines, on the system’s website communicating the information back to the public. Many of the sites also provide public health news or information about the diseases that they target.

### Unique contributions to public health

Several of the participatory surveillance systems described above have already demonstrated their accuracy and sensitivity, their ability to provide more timely measures of disease activity, and their usefulness for addressing public health challenges such as identifying risk groups, assessing burden of illness and evaluating vaccination coverage and effectiveness, and informing disease transmission models [4-8,10,17-19].

### Accuracy and sensitivity

After the first season of ILI surveillance in the Netherlands (2003/2004), de Grote Griepmeting data were compared to the official ILI data collected by the Dutch Sentinel Practice Network of physicians [4]. The timing of the ILI epidemic closely matched the official data, peaking the same week. This initial observation was later replicated by comparing Gripenet in Portugal and de Grote Griepmeting in the Netherlands and Belgium to sentinel physician networks for the 2006/2007 influenza season [7]. The ILI incidence rate among Gripenet and de Grote Griepmeting participants was also closely correlated with ILI incidence data from the European Influenza Surveillance Scheme (EISS)). These observations have proven to be robust over time as well. A comparison of data from five seasons of de Grote Griepmeting with the Dutch Sentinel Practice Network data found that Pearson correlations for each seasonal epidemic ranged from 0.69 to 0.90 [17].

Australian data collected by FluTracking from 2007 to 2009 were compared temporally with laboratory influenza antigen test data as well as ILI reports from emergency departments [20]. All three systems detected peak incidence concordantly, with no more than a week difference between them. This finding suggests that all systems are monitoring the same condition. In the UK, Flusurvey data also are highly correlated with the ILI incidence estimates reported by the Health Protection Agency (Pearson correlation: 0.71; 95% Confidence Interval: 0.44-0.87) [9]. The 2012/2013 influenza season data collected by Flu Near You for the United States has been compared with data collected by the CDC’s sentinel influenza network and Google Flu Trends [21]. While Flu Near You data followed the curve of the CDC data closely, data for more years and more participants are needed to verify these preliminary findings.

These studies have also identified the high sensitivity of participatory surveillance. The main difference between ILI incidence in de Grote Griepmeting versus sentinel surveillance was the amplitude; de Grote Griepmeting incidence rate was 10 times higher throughout the entire observation period [4]. This was again found to be true in Belgium and Portugal, though the degree varied by country [7]. Importantly, the definition for ILI was the same for all three countries in the participatory surveillance system, but not in the EISS system, making a true ILI comparison between the Netherlands, Belgium and Portugal challenging. One of the reasons for this difference in the magnitude between the systems may be country-specific variations in the likelihood of going to the physician. Nonetheless, this finding suggests that ILI incidence rates observed in participatory surveillance systems may be closer to the true incidence of ILI in these populations [7].

### Timeliness

The near real-time availability of data is currently a major strength of participatory surveillance systems. All healthcare-based surveillance systems can be slow because of persons waiting to seek treatment. While electronic health records will change this, additional delays occur due to manual reporting at various administrative levels within systems. Further delays occur in laboratory-based systems, which depend on sample collection and testing prior to notification. In participatory surveillance, there may be very little delay because those who are sick can report their symptoms directly to the system and their information can be assessed, aggregated and visualized almost instantly. This gain in time between the onset of disease and its reporting is a considerable advantage of participatory surveillance systems because it increases the possibility of detecting warning signals before traditional healthcare-based systems, though this is conditional on maintaining a sufficiently high rate of participation.

Some of the participatory surveillance systems have also demonstrated that the ILI signal is slightly earlier than in traditional surveillance systems. This has been exemplified by the need to lag their data in order to strengthen associations with traditionally collected data. While five seasons of de Grote Griepmeting data correlated well with the Dutch Sentinel Practice Network data, the correlation was strongest with a one week lag, suggesting that de Grote Griepmeting may detect changes in ILI incidence one week earlier than the traditional sentinel network [17]. Similarly, in Italy and Australia, Influweb and Flutracking data captured the H1N1 pandemic peak one week before the sentinel physician network [8,10]. A possible explanation for the earlier signal in both of these examples is that a sick individual does not go to the doctor on the first day of illness, but many participants in de Grote Griepmeting and Influweb report illness on the same day that they started feeling sick.

### Addressing public health questions

Surveillance data is used to address important questions related to control and prevention activities, such as identifying risk groups and monitoring intervention effectiveness. Here participatory surveillance also has much to offer. Influenzanet, for example, has consistently shown that public transportation does not increase the risk of developing ILI relative to driving a car, riding a bicycle or walking as a primary mode of transportation [5]. This result has been observed in the majority of the participating countries (the Netherlands, Portugal, Italy, United Kingdom, Sweden, France, and Spain) for all the seasons the systems have been in operation.

The UK Flusurvey data were used to estimate the effectiveness of the 2010/2011 influenza season vaccine [22]. Vaccination was associated with reduced ILI incidence, with an estimated vaccine effectiveness of 52% (95% CIs, 27%-68%) [22]. It was also associated with reduced absenteeism, especially for those between 25–64 years of age, with 4.1% of the vaccinated participants reporting taking time off work compared to 11.6% of the unvaccinated. Furthermore, vaccinated absentees were away from work for a significantly shorter period of time compared to the unvaccinated persons.

FluTracking data have also been used to estimate vaccine effectiveness [23]. Vaccine effectiveness was estimated to vary substantially across different years, from approximately 21% in 2007 and 23% in 2008 to essentially 0% in 2009, most likely due to the appearance of H1N1. As with estimates from other surveillance systems, these estimates may be sensitive to system-specific biases including participation bias, small sample size, and the fact that not all ILI cases are influenza.

In addition, FluTracking data have been used to explore vaccination coverage in participants, including specifically those with patient contact [24]. By the end of 2009, 28% of FluTracking participants and 41% of those who worked face-to-face with patients had received the pandemic vaccine. FluTracking was able to monitor this as it changed, with coverage increasing to 65% of participants and 78% of persons with patient contact by the end of 2010.

Healthcare-seeking behavior has been monitored in all Influenzanet countries. In the 2006/2007 season, 25% of all participants with ILI in the Netherlands, 45% of all participants with ILI in Portugal and 76% of all participants with ILI in Belgium visited a healthcare professional [7]. Specifically in Italy, 55% of participants who reported ILI symptoms phoned a physician while only 4% visited a physician [8], suggesting that almost half of all ILI cases have no contact with healthcare professionals and only a very small proportion are available for potential testing. Data from the UK Flusurvey during the 2009 H1N1 influenza epidemic showed that adults were 50% less likely to get medical attention than children [18]. This data allowed the authors to estimate that there were 1.1 million symptomatic influenza cases, a number 40% greater than the Health Protection Agency’s estimate of 780,000 cases (based on ILI physician consultations and internet- and telephone-based National Pandemic Flu Service). This estimate implies a 35% lower case fatality rate than previously estimated, as case fatality rates are dependent on the total number of cases detected.

### Strengths and weaknesses of participatory surveillance systems for infectious diseases

As discussed, participatory surveillance systems can be more sensitive and timely than traditional systems. In addition, these systems are independent from healthcare-seeking behavior biases and are less costly, more flexible and more scalable than tradition healthcare-based surveillance. However, participatory surveillance systems also offer unique challenges: there may be biases associated with the population that chooses to participate, adjusting for confounders may be complicated, reliance only on syndromic definitions of disease limits their specificity, and ensuring consistent participation is difficult.

**Figure 1:**
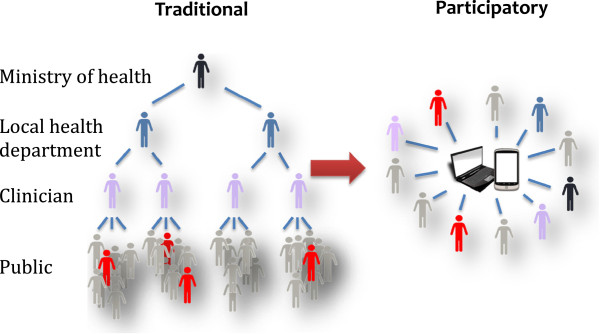
Comparison between traditional healthcare surveillance and participatory surveillance.

Traditional healthcare-based surveillance systems rely on sick people seeking medical care (Figure 1). Many people do not seek care for a variety of reasons, some related to disease severity, but others related to socio-demographic differences which introduce inherent biases [25,26]. These systems also require that the symptoms are attributed to the correct etiology, often aided by seeking laboratory confirmation, and that the relevant information is reported to the system [27]. Taking this entire process into consideration, it is easy to see why the number of cases reported in traditional healthcare-based surveillance methods is an underestimation of the true disease burden in the population. This is precisely where participatory surveillance systems can be used as an additional, supplemental data source to have a more comprehensive estimate of disease burden.

From a technical point of view, data collection in participatory surveillance systems is also easier and more streamlined than in traditional healthcare-based surveillance systems as it can be accomplished via a simple Internet-based form that is readily accessible from many locations. This also decreases the costs associated with operating a participatory surveillance system. Having all aspects of the system integrated, including participant recruitment, questionnaires, case definitions, analysis of the data and presentation of the results, increases the system’s flexibility. This means that almost any or all of the components of the system can be changed without disturbing the system’s overall functionality.

Scalability is another technical strength associated with participatory surveillance systems. The amount of resources (time, computation, finances and personnel) needed to expand or change a participatory surveillance system is minimal in comparison to those needed to expand a healthcare-based surveillance system, as no additional personnel or specific training of existing personnel is needed.

At the same time participatory surveillance systems have limitations, including participant populations that may not be representative of the general population. A majority of participants are women [9,17,20,28]. Children and the elderly tend to be underrepresented as the elderly and young are the least likely age groups to use the Internet [4,8,11,17,18,22,23,28]. However, Flusurvey was able to show that its participants were similar to the general population in terms of risk group status (diabetes, asthma, other chronic lung disease, immune-compromised, chronic heart disease, other chronic diseases and pregnancy), with the exception of the 0–14 age group [9]. Asthma and diabetes rates in participants and the general population have also been shown to be similar in the Netherlands [4] and Belgium [29]. And most of the systems have evolved mechanisms to include more children and elderly, by allowing participants to also report for other members of their household. In 2007, FluTracking expanded to include everyone 12 years or older not just those over the age of 18. FluTracking also allows participants to report on behalf of household members of any age [11]. For Flu Near You, any residents of the US or Canada 13 years of age or older can register and participants can report on their own health as well as the health of household members of any age [14]. Dengue na Web also allows participants to register household members who can either report for themselves or have the registering participant report for them [15].

Participatory surveillance also has limitations about how much data can be collected from participants. The more complicated and long the survey becomes for participants the less likely they are to contribute information to the system [30]. This is particularly important for identifying and adjusting for potential confounders and risk factors, including but not limited to chronic illnesses, pregnancy and immune status. The more information that is available, the more accurate the estimates of burden can be.

Participatory surveillance systems are inherently syndromic as none of the systems incorporate any laboratory testing. They thus depend heavily on the syndrome definitions and reporting behavior. A number of different ILI definitions are in use by surveillance systems in general, and that is also true of the participatory systems. While no definitions are right or wrong, it is vital for them to be clearly defined and analyzed. It is also possible to use other, complementary data sources to bolster participatory surveillance data, for example using more detailed data to estimate the probability of reporting ILI symptoms given that a person has influenza [31].

There are other issues related to participation, which is critical to all functions of participatory surveillance. All systems have found that recruiting and maintaining participants is a substantial challenge. Reporting fidelity is quite variable, with some participants reporting only once or twice, others sporadically over time, and many every week [8,9,17,18]. Participation rates also relate to illness; first-time participants are more likely to be sick than repeat participants. Limited access to internet may also limit participation. However, even in places with limited internet access, internet-based surveillance systems show promise [32].

### The future of participatory surveillance

Participatory surveillance is a quickly growing and evolving field. One of the key aspects that needs to be addressed for participatory surveillance systems to gain greater acceptance and credibility in the field of public health is data validation. This can be accomplished by comparison to traditional surveillance systems, as is already being done, or by laboratory testing of biological samples from participants reporting symptoms.

In addition to collecting disease data, participatory surveillance systems are improving awareness of the infectious diseases they monitor. However, these systems have the potential to go beyond that and increase a layperson’s knowledge of public health and its importance for society. Communicating this kind of information effectively could result in a greater appreciation and understanding of public health in the general population.

A large number of consistent users who are geographically dispersed and of diverse age and risk groups are needed for participatory surveillance systems to work optimally. Systems need to continue recruiting new members and expanding to areas where the general population is not well represented so that they collect and disseminate the best information possible. Ideally, participatory surveillance systems should be integrated into healthcare-based systems to supplement data obtained from traditional sources because they can provide information about people who do not seek healthcare, data that is not otherwise available.

Integration with traditional healthcare-based surveillance systems is also important from a system’s sustainability perspective. Although participatory surveillance systems are low-cost in comparison with traditional systems, they are not free and need active support to continue. Besides integration with an already existing traditional healthcare-based system, there are different revenue sources that can be used to support participatory surveillance systems. These sources include advertising on the system’s webpage, and non-profit or corporate sponsorships. Before deciding on what financial support to accept, it is important to understand and anticipate how various funding sources will be perceived by the public and whether the system’s reputation will be altered based on what organization provides the support.

Currently, healthcare-based surveillance systems rely on obligatory reporting of diseases in a one-way system where information feeds into an institutional reporting hierarchy. This classical paradigm requires revision for a better fit with today’s technological and societal advancements. Participatory surveillance systems promote the exchange of information between people and public health professionals, with the potential to spark a new level of engagement in an individual’s as well as a community’s health.

## Competing interests

The authors declared that they have no competing interests.

## Authors’ contributions

OPW, JSB, RC, and MAJ participated in the conceptualization of the review and helped to draft the manuscript. All authors read and approved the final manuscript.
